# Integration of schistosomiasis control activities within the primary health care system: a critical review

**DOI:** 10.1186/s13071-019-3652-z

**Published:** 2019-08-07

**Authors:** Paul Bizimana, Giuseppina Ortu, Jean-Pierre Van Geertruyden, Frédéric Nsabiyumva, Audace Nkeshimana, Elvis Muhimpundu, Katja Polman

**Affiliations:** 1 Global Health Institute, Department of Epidemiology and Social Medicine, Faculty of Medicine and Health Sciences, University of Antwerp, Antwerp, Belgium; 2 Département des Sciences de la Santé Publique, Direction de la Formation, Institut National de Santé Publique, Bujumbura, Burundi; 3 Département de Médecine Communautaire, Faculté de Médecine de Bujumbura, Université du Burundi, Bujumbura, Burundi; 4 Département des Sciences de la Santé Publique, Institut Universitaire des Sciences de la Santé et de Développement Communautaire, Bujumbura, Burundi; 5 Centre for Neglected Tropical Diseases, Liverpool School of Tropical Medicine, Liverpool, UK; 6 Département de Médecine Interne, Faculté de Médecine de Bujumbura, Université du Burundi, Bujumbura, Burundi; 7 Programme National Intégré de Lutte contre les Maladies Tropicales Négligées et la Cécité, Département des programmes de santé, Ministère de la Santé Publique et de la Lutte contre le Sida, Bujumbura, Burundi; 8 Medical Helminthology Unit, Department of Biomedical Sciences, Institute of Tropical Medicine, Antwerp, Belgium

**Keywords:** Control measures, Health centre, Integration, Primary health care system, Review, Schistosomiasis

## Abstract

**Background:**

Schistosomiasis is a chronic disease linked to poverty and is widely endemic, particularly in sub-Saharan Africa. For decades, the World Health Organization has called for a larger role of the primary health care system in schistosomiasis control, and its integration within the routine activities of primary health care facilities. Here, we reviewed existing studies on the integration of schistosomiasis control measures within the primary health care system, more precisely at the health centre, and we analysed their outcomes.

**Methods:**

An online search of studies published *via* PubMed and Embase databases was carried out until December 2017. Keywords were used to identify articles related to the integration of schistosomiasis control within the primary health care system, especially at the health centre level. Studies on integration of the following control measures were included: diagnosis and treatment, supplemented or not with (i) health education; (ii) snail control; and (iii) clean water supply and sanitation. A qualitative review was undertaken. To conclude on the effectiveness of an intervention, intermediate outcomes (knowledge, attitude and practice, coverage, access to health care) and distal outcomes (prevalence, incidence, mortality) were considered, and pre/post-intervention results were compared.

**Results:**

Of 569 records found, 11 met the inclusion criteria. Studies were classified in three groups, according to the control measures they included. Integration of diagnosis and treatment, and health education in the first group resulted in an improvement of knowledge level of care providers, access to health care and health care seeking behaviour of the community. However, no positive effect was observed on the knowledge level of symptoms and modes of transmission at the community level. Most studies in the second group (with snail control as additional measure) and the third group (with clean water supply and sanitation as additional measure) showed a positive effect on schistosomiasis prevalence and incidence post-intervention, independent of the additional control measures implemented.

**Conclusions:**

The results of this review suggest a positive impact of integration of schistosomiasis control within the primary health care system. However, more robust studies are needed, especially in resource-limited regions, for conclusive evidence on the effectiveness and the sustainability of this strategy.

## Background

Schistosomiasis is a parasitic disease acquired through contact with contaminated water, with debilitating and chronic complications [1]. Sub-Saharan Africa is the most affected with more than 90% of the total burden [2]. Estimations indicate that 207 million people are infected [2, 3] and more than 800 million are at risk [3]. In addition, schistosomiasis is responsible for an annual loss of 4.5 million disability-adjusted life years (DALYs) [4, 5] and 200,000 deaths every year [6]. School-age children are the most vulnerable group, with severe consequences for physical and cognitive development [7, 8].

For several decades, schistosomiasis prevention and control has been essentially based on vertical programmes organised and coordinated at the national level, with the support of donor organisations [9, 10]. Within these programmes, mass drug administration (MDA) campaigns delivering praziquantel (PZQ) to populations at risk, especially school-age children [11], have been organised annually or biannually. They have not been integrated within the health care structures but run as interventions in parallel with the routine prevention and control activities performed by the primary health care (PHC) structures [12].

In 1993, the World Health Organization (WHO) called for a larger role of the PHC system in schistosomiasis control [13], and its integration within the routine activities of PHC facilities. The components of this integrated control were defined as (i) health education, (ii) diagnosis and treatment (D/T), (iii) promotion of clean water supply, (iv) sanitation and (v) control of snails [13], with an emphasis on D/T [13]. This 1993 WHO strategy on integrating schistosomiasis in the PHC system, was reconsidered by the WHO in 2004 [14], and by others in 2010 [15, 16]. Recently in 2017, disease surveillance was added by the WHO [17]. The need to integrate these control measures within the PHC system has also been proposed as a response to the global call for schistosomiasis elimination [17]. Although successes have been reported with MDA [18–20], there is also increasing evidence that MDA alone will not be sufficient for achieving disease elimination [18–20].

So far, only a few studies have been performed on the integration of schistosomiasis control measures within the PHC system, more precisely at the health centre (HC) level. In this article, we review the existing studies and analyse the outcomes.

## Methods

### Search strategy and selection criteria

This review was carried out to identify studies on the implementation of schistosomiasis control in the PHC system, more precisely at the HC level, published up to December 2017 and available online, and where intermediate and/or distal outcomes [21] were available.

We performed computer-aided searches of the PubMed/Medline and Embase electronic online databases. Keywords used in PubMed/Medline were: (((integration OR control)) AND (schistosomiasis)) AND (“health centre” OR “Primary health care” OR PHC), while those used in Embase were: ((schisto* AND (integrat* OR control)) AND ((Health centre) OR (Primary health care) OR PHC). Only articles written in English or French were considered. In addition, bibliographies of published studies were screened to find additional sources of data.

**Fig. 1:**
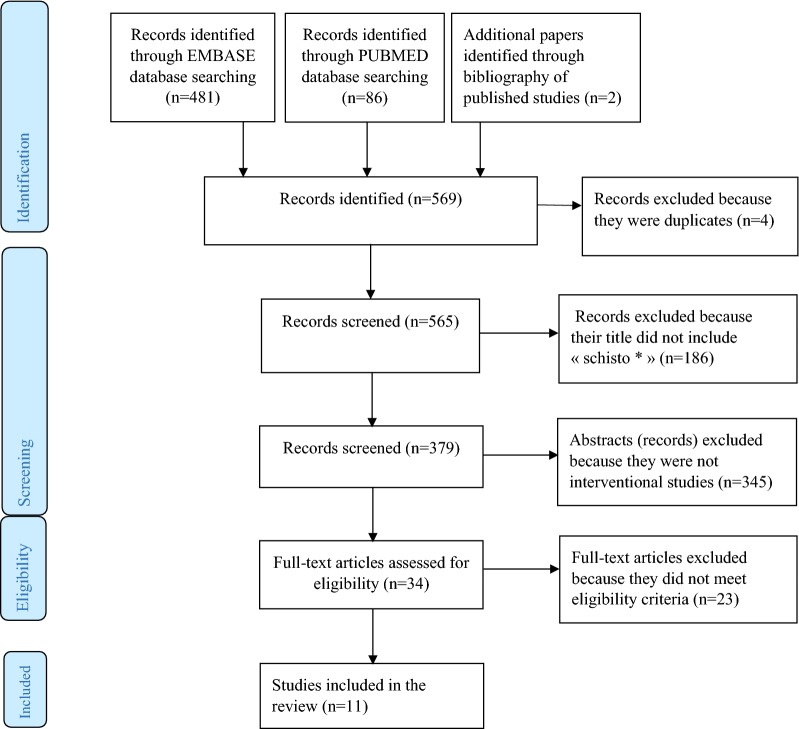
Flow diagram. The different steps in the selection process of studies included in this review

Subsequently, the title, abstract and full text were screened to identify articles that fulfil the inclusion criteria. Duplicates were excluded. For the title, only articles that contained “schisto*” were selected. The abstracts were screened, and only those describing an intervention were retained. For these studies, full papers were retrieved and further screened. Only studies including D/T at least, or supplemented with one or more of the following schistosomiasis control measures: (i) health education, (ii) snail control, and (iii) clean water supply and sanitation [13], in line with the 1993 WHO schistosomiasis control integration strategy [13], as inclusion criteria, were selected. The flow diagram of the literature search strategy is shown in Fig. 1.

From studies fulfilling the inclusion criteria, the following data were recorded: names of first author and year of publication; setting and duration of the intervention; *Schistosoma* species; control measures; population targeted; results pre/post-intervention; classification group; intervention effect.

### Assessment of the study outcomes

Studies were classified in groups, according to the control measures they included. Group one: studies that reported on D/T and health education. Group two: studies that reported on D/T, health education and snail control. Group three: studies that reported on D/T, health education, snail control, and clean water supply and sanitation. To conclude on the effectiveness of an intervention, different outcomes were considered and pre/post-intervention results were compared.

Two categories of outcomes were assessed: intermediate outcomes and distal outcomes [21–23]. The following intermediate outcomes were considered: (i) knowledge, attitude and practice (KAP) related to schistosomiasis of care providers (staff in charge of consultations and referrals, as a result of training) or targeted populations (as a result of health education); (ii) coverage (of D/T) and (iii) access (i.e. availability and financial accessibility) to health care. Distal outcomes were prevalence, incidence and mortality, due to schistosomiasis in the targeted population [23]. Table 1 shows in detail the different schistosomiasis control measures to be integrated within the PHC, at the HC level and different related outcomes.

**Table 1:** Linkage between different outcomes related to different control measures to be integrated within the primary health care system, at the health centre (HC) level

Outcomes	Household level	Community level	Health centre level
Proximal outcomes^a^			
Diagnosis and treatment (HC-based)^d^	Improved health care-seeking at HC	Community ownership/community participation to improve the disease related situation directly and/or *via* health committee; referral of suspicious cases to the HC for early diagnosis and treatment (D/T)	Staff ownership; early detection of positive cases; treatment of all positive cases; referral of complicated cases
Health education^e^	Early recognition of signs and symptoms; improved health care-seeking at HC	Community ownership/community participation to improve the disease related situation directly and/or *via* health committee; early recognition of suspicious cases; referral of suspicious cases to the HC for early D/T	Staff ownership; early detection of positive cases; treatment of all non-complicated cases; referral of complicated cases
Snail control^e^	–	Community ownership/community participation to improve the disease related situation (by regular contribution to the mollusciciding activities) directly and/or *via* health committee; improved environment	Reduction of snails in water used by people (by mollusciciding activities); improved environment (by regular mollusciciding activities);
Clean water supply and sanitation^e^	–	Community ownership/community participation to improve the disease-related situation by behaviour change (avoiding contact and defecating/urinating in/near water, use of latrines) and direct contribution to the construction activities; improved environment	Staff ownership; improved environment
Intermediate outcomes^b^	Improved knowledge, attitude and practice; improved coverage in D/T; and improved access to health care (i.e. availability and financial accessibility)	Improved knowledge, attitude and practice; improved coverage in D/T; and improved access to health care (i.e. availability and financial accessibility)	Improved knowledge, attitude and practice; improved coverage in D/T; and improved access to health care (i.e. availability and financial accessibility)
Distal outcomes^c^	Reduction in schistosomiasis prevalence; reduction in schistosomiasis incidence; and reduction in schistosomiasis-related mortality	Reduction in schistosomiasis prevalence; reduction in schistosomiasis incidence; and reduction in schistosomiasis-related mortality	Reduction in schistosomiasis prevalence; reduction in schistosomiasis incidence; and reduction in schistosomiasis-related mortality

^a^Proximal outcomes are different according to the control measure considered and the levels

^b^Intermediate outcomes are identical, regardless of the control measure considered and the levels. They are all common consequence/result of different proximal outcomes, which interact with each other, in synergistic way

^c^Distal outcomes are identical, regardless of the control measure considered and the levels. They constitute common consequences/results of different intermediate outcomes, that interact with each other, in synergistic way. They constitute the health impact of an intervention (programme activities)

^d^Diagnosis and treatment (HC-based) is a prerequisite control measure at the health centre level, for the integration of any other control measure

^e^Health education, Snail control and Clean water supply and sanitation are different control measures to be integrated within the primary health care system, especially at the health centre

When both distal and intermediate outcomes were available, distal outcomes were analysed. When distal outcomes were missing, intermediate outcomes were taken into account. When available, results of statistical analysis were reported (comparison pre/post-intervention, *P* < 0.05). In their absence, quantitative pre- and post-intervention results were compared, and when post-intervention results improved, it was concluded that the intervention had a positive effect, regardless of the improvement level. For post-intervention results related to KAP outcomes, more than 50% of the targeted subjects had to fulfil criteria that were considered as relevant (by the authors) to conclude that the intervention had a positive effect [24]. If quantitative results were not available, the qualitative appraisal of the authors of the respective studies was taken into account.

## Results

In total, 569 titles were identified in all databases explored. After titles and abstracts were screened, 34 full texts were analysed, from which 11 studies were selected for the review (Fig. 1).

Out of these 11 studies, four were realised in West Africa (Senegal, Mali), three in Asia (Saudi Arabia), two in East Africa (Burundi), one in South America (Brazil) and one in Southern Africa (Botswana). Six papers were published between 1990 and 1995, three between 2000 and 2003 and two before 1990. The oldest publication was from 1988 and the most recent from 2003. All studies were before-and-after studies [22], without a control group. The main results are summarized in Table 2.

**Table 2:** Characteristics of included studies, control measures included and results pre/post-intervention

Author and year	Country and duration of the intervention	*Schistosoma species*	Control measures	Population targeted	Results pre/post-intervention	Classification group	Intervention effect
Landouré et al. (2003) [25]	Mali, 1 year (1986–1987), evaluation of integration 10 years later	*S. haematobium*; *S. mansoni*	Diagnosis and treatment^a^; health education^b^ and community mobilisation	General population	Knowledge of symptoms and treatment: *S. haematobium*: -/80%; *S. mansoni*: -/69% Praziquantel available, but some shortages in some health centres Cost for praziquantel relatively high	One	+
Sow et al. (2003) [26]	Ndombo village, Northern Senegal, 7 years (1994–2000)	*S. mansoni*	Diagnosis and treatment^a^; health information^b^; health education^b^ on health-seeking	General population	Knowledge: symptoms: 0%/54.2%; mode of transmission: 0%/43.5%; both combined: 0%/29.5% Health care seeking behaviour at health centre: 0%/ 92%	One	±
van der Werf et al. (2002) [27]	Saint Louis Region, Northern Senegal, 4 years (1995–1999)	*S. mansoni*	Diagnosis and treatment^a^; health education^b^ at the community level	General population	Knowledge of symptoms and treatment: *S. haematobium*: -/100%; *S. mansoni*: -/94% Praziquantel available Cost of praziquantel relatively cheap	One	+
Ageel & Amin (1997) [28]	Gizan Region, Saudi Arabia, 5 years (1990–1996)	*S. haematobium*	Diagnosis and treatment^a^; snail control^c^; health education^b^ of the population	General population	Community participation Improved coverage in diagnosis and treatment: 60% (1990)/90% (1996) Overall prevalence: 1.2% (1990)/0.3% (1996)	Two	+
al Moagel et al. (1990) [29]	Riyadh Region, Saudi Arabia, 5 years (1984–1988)	*S. haematobium*; *S. mansoni*	Diagnosis and treatment^a^; mollusciciding^c^; health education^b^	General population	Improved coverage in diagnosis and treatment: 10688 cases (1984)/106579 cases (1988); overall prevalence: 9.3% (1984)/0.6% (1988)	Two	+
Brinkman et al. (1988) [30]	Office du Niger irrigation zones and the district of Bandiagara, Mali, 1 year (1986–1987)	*S. haematobium*; *S. mansoni*	Diagnosis and treatment^a^; health education^b^ of population; mollusciciding^c^	General population	Prevalence < 20% in villages targeted by the intervention: *S. haematobium*: 8.1% (1986)/46% (1987); *S. mansoni*: 36.7% (1986)/53% (1987) Prevalence of intensive infections < 5% in villages targeted by the intervention: *S. haematobium*: 23% (1986)/72% (1987); *S. mansoni*: 36% (1986)/50% (1987)	Two	+
Jarallah et al. (1993) [31]	Riyadh, Saudi Arabia, 3 years (1984–1986)	*S. haematobium*; *S. mansoni*	Diagnosis and treatment^a^; snail control^c^; health education^b^ of the population	General population	Overall prevalence: 13.2% (1983)/0.2% (1989) Prevalence of *S. mansoni*: 12.3% (1983)/0.1% (1989) Prevalence of *S. haematobium*: 0.9% (1983)/0.05% (1989) Prevalence in Saudians: 91.1% (1983)/32.6% (1989) Prevalence in non-Saudians: 8.9% (1983) /67.4% (1989) Dropout rate of patients under treatment: 54.4% (1987)/22.1% (1989)	Two	+
Coura et al. (1992) [32]	Peri-Peri, Brazil, 3 years (1984–1987)	*S. haematobium*; *S. mansoni*	Diagnosis and treatment^a^; basic health education^b^; basic sanitation; malacological^c^ control	General population	Improved coverage in diagnosis and treatment: 81.7% (1984)/91.8% (1987) Prevalence: 15.2% (1984)/4.4% (1987) (*P* = 0.01) Incidence: 10.9% (1984)/2.9% (1987) (*P* = 0.002) Cure rate: children: 72% (1984)/88% (1987); adults: 83.3% (1984)/94% (1987)	Three	+
Ali et al. (1989) [33]	Ngamyland, Botswana, 3 years (1985–1987)	*S. mansoni*	Diagnosis and treatment^a^; snail control^c^; health education^b^ and community awareness; water supply^d^; improved sanitation^d^	General population	Community participation Overall prevalence: ≤ 10% (survey in school children)/3.3% (survey in school children) Successful reduction in prevalence of *S. mansoni* infection in general population Infection intensity (> 100 eggs per gram): 5.4% (for the first survey)/0.5% (for the third survey)	Three	+
Engels et al. (1993) [34]	Bugesera, Bujumbura, Imbo-Sud, and Rusizi plain, Burundi, 3 years (1989–1992)	*S. haematobium*; *S. mansoni*	Diagnosis and treatment^a^; health education^b^ of school children and patients; snail control^c^; safe water supply^d^; construction of latrines^d^	General population	Improved coverage in diagnosis and treatment Rusizi plain: 970 cases (1988)/3584 cases (1991) Bugesera: 63 cases (1988)/180 cases (1991) Sustainability and affordability for the national health budget of the integration	Three	+
Engels et al. (1995) [35]	Bubanza, Bujumbura, Bururi, Cibitoke, Kirundo, Makamba, Burundi, 3 years (1989–1994)	*S. mansoni*	Diagnosis and treatment^a^; health education^b^ of school children and patients; snail control^c^; safe water supply^d^; construction of latrines^d^	General population	Apparent recovery of an integrated control programme in the primary health care, after civil unrest Sustainability of a schistosomiasis control programme which is integrated in the primary health care	Three	+

*Key*: +, positive; ±, low impact

^a^Diagnosis and treatment: integrated into routine care after training of the staff in charge of consultations and referrals. Within this control measure, the targeted population was the general population accessing health care facilities

^b^Health education: performed by staff in charge of consultations, referrals and in charge of hygiene and sanitation. The target population was patients, school children and the general population

^c^Snail control: integrated into routine activities after training of the staff in charge of hygiene and sanitation at the health centre. The target was water bodies in schistosomiasis endemic areas

^d^Clean water supply and sanitation: the integration in the routine activities implies that the staff in charge of hygiene an sanitation is always involved in the identification of hygiene and sanitation issues, in households within the health centre’s area of responsibility, and in the management of those which can be solved at their level. Otherwise, they must report identified and unsolvable problems to the administration, which had to find solutions. All control measures implied provision of necessary resources (drugs, laboratory tests, equipment and supply) and training of health care staff for the implementation of the needed activities

### Intervention effect analysis

#### Group one: D/T and health education

Group one consisted of three studies [25–27] which reported on intermediate outcomes only. In Senegal (2002) [27], the knowledge level of care providers in the HC post-intervention was much improved for both *S. haematobium* and *S. mansoni*. Results were similar but less impressive for the study in Mali (2003) [25]. Authors reported that PZQ was available and financially accessible in Senegal (2002) [27], while in Mali (2003) [25] some shortages were noted in some HC, with relatively high costs. Integration of D/T and health education was considered to be successful in Senegal (2002) [27] and satisfactory in Mali (2003) [25]. Another study in Senegal (2003) [26] reported improvement in knowledge level of symptoms and in health care seeking behaviour at HC, reaching 54.2 and 92% post-intervention, respectively. However, the knowledge level of modes of transmission, and both symptoms and modes of transmission at community level [26] was reported to be low.

#### Group two: D/T, health education and control of snails

Five studies belonged to group two. Four [28–31] of the five studies reported on prevalence only, while one [32] reported on prevalence and incidence. A significant decrease in prevalence (*P* < 0.05) and incidence (*P* < 0.05) was reported post-intervention in the study in Brazil (1992) [32]. The schistosomiasis prevalence decreased to less than one percent in three studies carried out in different regions in Saudi Arabia [28, 29, 31]. For a study in Mali (1988) [30], the outcome criterion was the number of villages with an infection prevalence of less than 20%. The number of villages which fulfilled this condition increased from 8.1% to 46% for *S. haematobium* and from 36.7% to 53% for *S. mansoni* post-intervention.

#### Group three: D/T, health education, control of snails, and clean water supply and sanitation

Three studies were categorised in group three. One study in Botswana (1989) [33] reported on schistosomiasis prevalence and showed a decrease in prevalence post-intervention. One study in Burundi (1993) [34] reported on D/T coverage and affordability and showed an improved coverage. The authors reported that the available health infrastructure had allowed the control strategy to be integrated to a high degree into basic health services enabling it to be sustained and making it affordable for the national health budget. The third study was a report [35] on the number of cases of schistosomiasis detected in each quarter-year in basic health services in six schistosomiasis endemic provinces of Burundi. These were targeted by integration of schistosomiasis control in the PHC facilities, from 1989 onwards. According to the authors, results showed the sustainability of the PHC integrated schistosomiasis control programme.

## Discussion

This review aimed to identify studies on the integration of schistosomiasis D/T and other control measures in the PHC system, especially at the HC level, and to evaluate their effects.

We identified 11 studies which were all published before 2003. The lack of more recent studies could be explained by the fact that the emphasis of schistosomiasis control strategies has since then been on MDA [36], following the adoption of World Health Assembly (WHA) resolutions 54.19 (2001) and 65.21 (2012) that set control and elimination goals for schistosomiasis, respectively. Due to its cost-effectiveness and feasibility in low-resource settings, this strategy became the mainstay of schistosomiasis control, and other less straightforward strategies were given less attention. Since the global call for schistosomiasis elimination and the increasing evidence that MDA alone will not be sufficient for achieving this goal [18–20], the integration of schistosomiasis-related interventions into broader health systems has been put back on the agenda. Integrating schistosomiasis control should ensure that the delivery of health services meets the needs of those living with schistosomiasis. Moreover, it has the potential to accelerate progress towards Universal Health Coverage and to advance the Sustainable Development Goals [17].

For the studies in group one (D/T and health education) all reporting on intermediate outcomes only, knowledge level of care providers, access to care and health care seeking behaviour of the community increased. However, no positive effect was observed on the knowledge level of symptoms and of modes of transmission at the community level.

The majority of studies from group two (with snail control as additional measure) and group three (with clean water supply and sanitation as additional measures) reporting on distal outcomes, showed a positive effect on schistosomiasis prevalence and incidence post-intervention, independent of the additional control measures implemented.

The positive effect on knowledge level of care providers following the training of the staff in charge of consultation and referrals on schistosomiasis, is supported by the literature [37–40]. The lack of a positive effect on knowledge at the community level may be explained by the complexity of public health interventions [40], such as health education, which may take a long time before achieving results.

The availability of resources for D/T, as well as financial accessibility for patients, are indispensable for improved access to care [15, 41]. In Senegal, the retail selling price of drugs was determined by the local health committee, a group of representatives chosen from the population, which may have had a strong influence on the relatively low costs of PZQ and hence the access to treatment [26].

The positive effect on health care seeking practice at HC as observed in this review may be linked to the availability of means for the D/T at the HC, encouraging the population to visit a HC since they know they will be treated [42].

All studies included (i) D/T and (ii) health education. Some studies included additional control measures, such as control of snails (group two) or control of snails, and clean water and sanitation (group three). For all groups, distal outcomes were similar. One could conclude that clean water and sanitation did not bring any added value. However, it should be noted that the majority of the studies in group two were conducted in settings with a relatively high socioeconomic level [28, 29, 31, 32], which is likely to have contributed to the overall positive effect on schistosomiasis prevalence and incidence. Full socio-economic development remains essential for sustainable control and possibly elimination of schistosomiasis and other poverty-related diseases.

Another important aspect is the political stability of the region in which the interventions are initiated [22]. Its absence can lead to the reduction and even total cessation of integrated control activities. However, Engels et al. [35] showed that integrated control at PHC, especially at the HC level, appears to be resilient, even after severe civil unrest of three years. In addition, a successful integration of schistosomiasis control measures at PHC level requires well functioning health systems [34, 43]. If the health system structure is already weak, it will only be further weakened, thereby not only hampering schistosomiasis control but also jeopardizing basic health care [11].

### Limitations

This critical review has demonstrated the scarcity of studies showing the impact of integration of schistosomiasis control within the PHC structures. None of the identified studies was a randomized control trial, or had a control group, which could have provided more solid evidence on the impact of the studied interventions. Some studies had missing pre-intervention or incomplete data [25–27, 35] and some did not have distal outcomes [25–27, 34, 35]. In addition, most studies had a limited duration and could therefore not provide sufficient evidence on the sustainability of the described interventions. There was also heterogeneity in study populations, methodologies and settings [22], which made comparison between studies difficult.

## Conclusions

The results as described in these studies support the idea of a larger role for the PHC system in schistosomiasis prevention and control, and its integration within the routine activities of PHC facilities. However, more robust studies are needed, both qualitative and quantitative, especially in resource-limited regions, to conclusively demonstrate the effectiveness of integration of schistosomiasis control in the PHC system. The emphasis of current global schistosomiasis control strategies is on MDA with praziquantel, but this will likely not result in sustainable control, let alone elimination of schistosomiasis. Integration of D/T and other schistosomiasis control measures into the PHC system offers clear opportunities to reduce the schistosomiasis burden in a sustainable way, thereby contributing to the Sustainable Development Goals.

## Data Availability

Data supporting the conclusions of this article are included within the article.

## Abbreviations

DALYs: disability-adjusted life years
HC: health centre
KAP: knowledge, attitude and practice
MDA: mass drug administration
PHC: primary health care
PZQ: praziquantel
WHO: World Health Organization
